# The Role of Mass Drug Administration of Antimalarials

**DOI:** 10.4269/ajtmh.20-0729

**Published:** 2020-07-02

**Authors:** Pedro L. Alonso

**Affiliations:** Global Malaria Program, World Health Organization, Geneva, Switzerland

Either intuition or empiricism must have led to the use of antimalarial drugs to both treat and prevent malaria, predating the identification of the malaria parasite and the mode of its transmission. Josep Masdevall in the XVIII century managed epidemics in Spain through the administration of compounds that included the bark of the cinchona tree. In more recent times, mass drug administration (MDA) was the first form of chemoprevention used against malaria in the early 1900s. Initially, under the term “quininization,” MDA was recommended in African and Asian countries, in combination with residual insecticides, to eliminate malaria during the “attack phase” of the Global Malaria Eradication Program.^1^ Since then, MDA has been widely used, particularly in Europe and Asia, where campaigns with primaquine are likely to have played a key role in the elimination of *Plasmodium vivax* from central Asia and China. Regardless of this very long history as a malaria strategy, MDA is not considered a core malaria intervention. Mass drug administration is defined as the use of a full therapeutic course of an antimalarial medicine, irrespective of the presence of symptoms or infection, to a specific population living in a defined geographic area, with the entire target population receiving the drug at approximately the same time, and treatment often repeated at intervals.^2^ This definition overlaps with other forms of chemoprevention, such as the targeting of pregnant women (intermittent preventive therapy in pregnancy), children living in areas with marked seasonality of malaria transmission (seasonal malaria chemoprevention), and infants living in areas of high to moderate transmission (intermittent preventive therapy in infants).

The WHO does not currently recommend the use of MDA for *P. vivax* malaria. Based on the evidence,^3^ it does, however, consider its use for *P. falciparum* malaria, with two distinct, complementary objectives or use cases.^2^ The first is to reduce transmission, with the intent of accelerating progress toward malaria elimination. Repeated rounds of MDA are given to reduce total parasite biomass. Synchronization of the intervention, with a high coverage of the entire population, is essential, as is the use of other malaria control tools and strategies, such as effective vector control and prompt diagnosis and treatment.^4^ The second use case for MDA is the rapid reduction of morbidity and mortality in epidemics and complex emergencies, when health systems are overwhelmed and unable to provide adequate malaria control. In such settings, MDA is used as an initial emergency measure; several rounds are implemented while efforts are made to strengthen access to case management and vector control. In this second use case, it is important to identify the population at risk for severe malaria and death to define the target groups.^4^

Today, the control of malaria relies on vector control as the core intervention to reduce transmission, combined with prompt and effective treatment of confirmed malaria cases to reduce case fatality. In recent years, different forms of chemoprevention have been introduced to prevent infections in high-risk groups, and thus prevent morbidity and mortality. These approaches are slowly gaining momentum, but, overall, coverage remains low.^5^ Mass drug administration has been used mainly in health emergencies, such as in the 2013–2016 West African Ebola crisis or the ongoing northeast Nigeria humanitarian crisis which has left two-thirds of health facilities completely or partially destroyed and some 3.7 million internally displaced people at risk of life-threatening diseases. However, with the exception of use in travelers to endemic areas, MDA and other forms of chemoprevention seem to carry negative connotations and meet with resistance. Several factors contribute to this, including the sense of failure from the use of chemoprevention during prior malaria eradication campaigns and the fear of inducing parasite resistance.

Global progress in malaria control has stalled in the last few years.^5^ We are probably seeing the limits of what can be achieved with the imperfect tools and limited financial resources available. There is thus a need to challenge the status quo if malaria’s contribution to the unacceptably high levels of under-five mortality is to be tackled. Doing the same thing over and over again will not allow us to get back on track to meet the internationally agreed targets of the Global Technical Strategy for Malaria 2016–2030.^6^

In response, the WHO and partners have called for the implementation of a High Burden to High Impact approach.^7^ Central to this approach is the use of local data to move away from a one-size-fits-all approach and rather to identify the optimal mix of interventions for particular subnational settings to maximize the impact of available resources.^8^

The use of malaria medicines for the prevention of infection—either in high-risk groups or for entire populations—remains one of the few options available and one whose full potential has yet to be realized. We thus welcome the generation of new data, as exemplified by the articles in this *AJTMH* supplement,^9^ as well as the review of old data, that will help the WHO strengthen evidence-based policy recommendations and lead to greater impact in endemic countries.
